# Perception of the Health Threats Related to the Consumption of Wild Animal Meat—Is Eating Game Risky?

**DOI:** 10.3390/foods10071544

**Published:** 2021-07-04

**Authors:** Katarzyna Niewiadomska, Małgorzata Kosicka-Gębska, Jerzy Gębski, Marzena Jeżewska-Zychowicz, Marianna Sułek

**Affiliations:** Institute of Human Nutrition Sciences, Warsaw University of Life Sciences (SGGW-WULS), Nowoursynowska 159c, 02-776 Warsaw, Poland; malgorzata_kosicka_gebska@sggw.edu.pl (M.K.-G.); jerzy_gebski@sggw.edu.pl (J.G.); marzena_jezewska_zychowicz@sggw.edu.pl (M.J.-Z.); marianna_sulek@sggw.edu.pl (M.S.)

**Keywords:** game meat, consumer segmentation, health risks, meat safety

## Abstract

Consumer interest in game meat has increased in recent years. Consumers appreciate its nutritional value but still have many concerns. Based on data from a quantitative study conducted in the group of 450 purposively selected Polish respondents declaring to consume the game meat, consumers were segmented concerning the perception of health risks associated with its consumption. Three separate clusters were identified using hierarchical cluster analysis: Indifferent (42%), Fearful (30%), and Selective (28%). The clusters differed significantly in the perception of the role of game in their lives and taking actions to mitigate the health risks associated with its consumption. In addition, their socioeconomic profiles were significantly different. The Indifferent segment—significantly more often than the other segments—believes that game has a positive impact on health, and the way to counteract the health risks is to not eat raw meat. The Selective segment attaches great importance to the choice of consumption place as a warranty of access to safe meat. The Fearful segment is willing to pay more for good quality meat and search for information. The results proved that the game consumers are not a homogenous group. Recognizing the differences can indicate a path for the traders to efficiently meet the consumers’ expectations and needs.

## 1. Introduction

Many have noted the increase in public interest in game meat consumption [1,2,3]. However, there are consumers who do not accept this type of meat for sensory and ethical reasons, as well as concerns about its safety and health effects [4].

When considering game meat consumption, two types of meat should be taken into account: hunted wild game meat (HWGM) and the meat of farming wild animals, both valued by consumers despite their different physicochemical characteristics [5,6,7,8]. It has been proved that the nutritional value of meat derived from wild animals living freely is greater than from the farming ones [9]; however, the parameters of the HWGM is depending on many aspects of the animal life and obtaining circumstances [10]. The consumers perceive wild animal farming as lowering the prestige of game meat and denying the product’s “naturalness” or the opposite, as emphasizing the quality and safety of meat [11]. It may also be an expression of care for the natural environment and the animal population, which is of particular importance in the case of rare species [11,12,13]. The studies prove that consumers tend to show little knowledge whether the meat is derived from hunted or farmed animals [6,14,15], and also, they are not interested in investigating the topic [16].

Considering consumers’ changing expectations and needs, it cannot be ignored that the importance of a given factor may increase in the future. However, now in European statistics, game meat is included as one type of meat, regardless of the obtaining method (farming or wild), due to its low consumption compared to other types of meat [3].

Game meat is generally considered to be expensive [17], exotic and luxurious meat [18], and seldom available on the market [19]. It also has a positive image as an “organic” product [20].Many consumers highly appreciate game for its nutritional value, as well as for its taste, aroma, and natural origin [2,21,22,23,24]. Among the factors influencing the choice of game are mentioned its availability, price, and quality [3,25]. The factors that determine the quality of game meat include its microbiological safety, ethical obtaining practices (animal welfare), and also healthiness, and the sensory profile (aroma, flavor, taste, and overall eating satisfaction) [21,26].

Meat derived from wild animals is described as a high nutritional value product, due to the low content of fat, beneficial fatty acid composition, and favorable amino acids configuration [27,28,29,30]. That makes it an attractive source of proteins, considering the need to ensure high nutrition quality and current researches showing that excessive meat consumption, especially red meat, may have a negative impact on human health [31,32]. The research on game meat has also shown that the aspects of the influence on the body weight and the limited fat content in the meat are crucial motives of the choice of game [27].

The safety of the consumption of the game, as in the case of other types of meat, is an important issue determining consumers’ interest [23]. However, it is worth mentioning that game meat is generally perceived as a safe product [29]. Significant threats directly related to the consumption of game include zoonoses [33], microbiological contamination [34,35,36], and also the presence of heavy metals [37], bullets/pellets residues [38], pesticides, antibiotics, and hormones [39]. Studies from Europe, Australia, North America, and South Africa show that the possibility of contracting zoonoses states the greatest risk for game meat consumers [1,2,24,39].

During the review of the literature, it was found that the consumers’ perception of the game meat quality is of interest to researchers [6,22,40]. The surveys on game meat safety have investigated the attitudes of consumers from different countries [2]. They have included the opinions of different stakeholders in the game meat industry [41] and also compared the opinions of people eating and not eating wild animal meat [42]. To understand the consumers’ concerns about game safety better, in the study, we have analyzed the particular threats and their impact on the consumers’ behaviors. So far, a segmentation of game consumers based on the health risk perception has not been performed. Thus, our results may arouse the interest of the researcher and game producers interested in the growth of the game market.

An original issue raised in this study is the verification of the kind of hazards that are of greatest concern to consumers. The results not only illustrate how safety is perceived in general but also indicate areas where special attention is required from producers and traders. Actors in the market and retailers wishing to sell the game should know what health concerns potential game consumers have. When preparing promotional activities, they should be able to show consumers that their ideas are often stereotypical and that the product available on the market is safe and does not contain contaminants that could pose a health risk to consumers.

The following three aims of this paper were formulated: (1) To determine consumer segmentation based on their perception of health risks related to the consumption of games; (2) to identify the differences between the segments due to consumers’ views on game meat, including its health benefits; (3) to discover what action consumers take to reduce the level of risk of game consumption.

## 2. Materials and Methods

### 2.1. Ethical Approval

The study was conducted according to the guidelines of the Declaration of Helsinki and approved by the Ethics Committee of the Faculty of Human Nutrition and Consumer Science, Warsaw University of Life Sciences, in Poland (Resolution No. 03/2016). Informed consent was provided by participants.

### 2.2. Study Design and Sampling

The data have been collected in a wide nationwide quantitative survey entitled, “Influence of selected conditions on consumer behavior towards game meat”. The research covered many aspects of game consumption. The following factors were investigated, including the impact of selected individual consumer characteristics and external environmental characteristics, as well as characteristics related to meat quality on consumers’ decisions about purchasing and consuming game. A survey was conducted by a professional market research agency in 2016, using the computer-assisted telephone interview method (CATI). The survey was addressed solely to those who declared to consume game meat. The sample of 450 Polish adults aged 25 or older was gathered using random dialing and validation of telephone numbers. The group reflected the demographic structure of the Polish population in terms of age and gender, following the public data of the Central Statistical Office (GUS). More information on the survey and sampling has been provided in the article discussing the consumers’ motives of choice of game meat [25]. Although the data was obtained using the same questionnaire, different aspects of the subject have been presented in the two papers. Also, other questions provided to the considered population were analyzed that were not reported in the previous article.

The size of the study sample results from the selection aimed solely at game consumers. However, the study sample is comparable to similar consumer research on meat consumption in other countries [2,43].

### 2.3. Questionnaire

The questionnaire contained questions about (1) health risk factors associated with the consumption of game; (2) attitudes towards game meat; (3) buying and eating behavior that ensure safety and mitigate health risks; and (4) opinions on the sources of information used to extend knowledge about the safety of game meat and its impact on health.

The opinions on health risk factors associated with the consumption of game were assessed using the question “What are you afraid of when you eat game meat?” The risk factors included: infection with parasites, infection with zoonoses, increase in cholesterol levels, gaining weight, and intake of heavy metals remaining in meat after a shot. They were selected based on a literature review [40,41], as well as the results of a pilot study. Respondents reported their opinions using a five-point scale ranging from “there is no such risk” (1) to “the risk is serious” (5).

The attitudes towards game meat were assessed using seven statements: (1) Game is a readily available product that can be found in many stores; (2) I do not accept hunting as a method of obtaining game; (3) Game meat and products provide good value for money; (4) Game meat has a positive effect on my health; (5) I value game meat for its natural origin; (6) In my home, game dishes are prepared only for special occasions and festivities; (7) I took the tradition of eating game meat from my family home. Respondents reported their opinions by choosing “Yes” or “No” option.

The buying and eating behavior that ensure safety and mitigate health risks were assessed using seven statements: (1) I eat game only in a trusted eating place; (2) I only buy/eat game that has been tested by a veterinarian; (3) I only buy game from a reliable source; (4) I am willing to pay more for better quality meat; (5) I only eat game at home; (6) I do not eat raw meat; (7) I seek information on game. Respondents expressed their opinions choosing “Yes” or “No” option.

The opinions on the sources of information used by participants to extend their knowledge about the safety of game meat and its impact on health were measured using a five-point scale ranging from “I do not use this source at all” (1) to “I use this source most frequently” (5). The following sources of information have been included in the questionnaire: culinary programs (e.g., TV, radio); the Internet (e.g., from food portals); producers’ websites; websites of the stores; family and friends; doctors and nutrition specialists; sellers; packaging; advertising (e.g., TV, press, billboard); store leaflets; press articles; culinary books; and restaurant service staff.

### 2.4. Statistical Analysis

The frequency analysis and the contingency tables were used to analyze the data. Study participants’ segmentation was performed using a hierarchical cluster analysis [44]. Consumers were segmented in terms of their opinions on risks associated with game consumption. A dendrogram was used to select the number of clusters, and the correctness of cluster separation was confirmed by CCC (Cubic Clustering Criteria), Pseudo F, and Pseudo T2 statistics.

The profiling of the clusters was carried out using the following sets of variables: the attitudes towards game meat, the buying and eating behavior that ensure safety and mitigate health risks, the opinions on the sources of information about the safety of game meat and its impact on health, and demographic characteristics.

The one-way analysis of variance ANOVA with post-hoc Waller–Duncan K-ratio *t*-test and Chi-square test was used to find significant differences between the groups. Significance was set at *p* < 0.05 for all analyses [45]. The statistical package SAS 9.4 was used for statistical analysis (SAS Institute, Cary, NC, USA).

## 3. Results

### 3.1. Characteristics of the Study Sample

The sociodemographic characteristics of the study sample, including gender, age, education, place of residence, professional situation, household size, self-reported financial situation, are presented in Table 1. The total sample comprised more men (58.7%), people aged 25–34 (32.0%), those with secondary education (77.3%), living in an urban area (54.7%), full-time employees (75.6%), people living in smaller households (three persons or less—54.2%), and those reporting that they could afford everything and could save some money (56.2%).

### 3.2. Characteristics of the Identified Clusters

As a result of cluster analysis, three groups (clusters) of consumers of game meat were identified (Table 2). Cluster 1 (“Selective”) comprised 125 respondents (27.8% of the study sample) and is characterized by the highest diversity of opinions on health risk factors associated with the consumption of game. They were afraid of infection with parasites and zoonoses the most, and they had the least fear associated with gaining weight and increasing their cholesterol level. In cluster 2 (“Indifferent”), comprising 190 respondents (42.2%), the risk of all factors was assessed as low. However, in cluster 3 (”Fearful”), comprising 135 study participants (30.0%), all factors were assessed as more risky compared to the other clusters.

There were differences between identified clusters after taking into account the socio-demographic characteristics (Table 1). The “Selective” group comprised more people aged over 55, with secondary education, part-time workers, but also students, those having four-person households, and reporting worse financial situation. The “Indifferent” cluster comprised the most men, people aged 45–54, those living in rural areas, and pensioners, whereas there were the least respondents from one-person households. The “Fearful” group comprised more women, people aged 25–34, with higher education, from urban areas, full-time workers, and those reporting a good financial situation.

### 3.3. Attitudes towards Game Meat

The respondents declared the highest level of compliance with the statement that they value game primarily for its natural origin. Approximately 80% of the study sample agreed that “they do not accept hunting as a method of obtaining meat”, and that “in their homes, game dishes are prepared only for special occasions and festivities”. Only slightly fewer people said that “game has a positive effect on health” (77.6%), and “game meat and products are value for money” (76.0%). The majority of the respondents did not agree that the tradition of eating game meat was taken from their family home (Table 3).

Most of the “Selective” group agreed that game meat and game products are valued for money. At the same time, the least of them reported that game dishes in their homes are prepared only for special occasions and festivities. When compared to other clusters, the largest number of people of the “Indifferent” group indicated that game had a positive effect on health, and that they took the tradition of eating game meat from the family home. However, the least of “Indifferent” indicated that game is worth its price and is valued for its natural origin. Most of the “Fearful” group did not accept hunting as a method of obtaining game. At the same time, they valued game meat for its natural origin. Moreover, they informed that game dishes are more often prepared at home for special occasions or festivities. Compared to the “Selective” and the “Indifferent” group, fewer people in the “Fearful” group agreed that game has a beneficial impact on health. The identified clusters did not differ regarding the opinion on the availability of game (Table 3).

### 3.4. Behavior Ensuring Safety and Mitigating Health Risks

To ensure safety, most of the respondents reported that they do not eat raw meat (95.1%), that they only eat it at home (86.4%), or that the meat is tested by a veterinarian (86.2%). Fewer people reported consuming game only in a trusted eating place (52.0%) and buying it from a reliable source (62.9%) (Table 4).

Most people from the “Selective” cluster indicated a trusted eating place and home as the places where they consumed game to ensure their safety. People from the “Indifferent” group declared that buying and eating game meat tested by a veterinarian most often. Compared to other clusters, much less, because only about half of them declared that they buy game from a reliable source. The “Fearful” group were characterized by the highest number of indications informing that they do not eat raw meat, want to pay more for better quality meat, and seek information on game. While the least of these people indicated that they eat/buy game tested by a veterinarian, from a reliable source, and/or in a trusted eating place (Table 4).

### 3.5. Sources of Information on the Safety and Health Benefits of Game Meat

The respondents’ opinions on using the sources of information about the game meat are presented in Table 5. The most frequently used sources were the sellers and packaging (labels), and then family and friends, store leaflets, and the Internet, i.g., pages dedicated to food issues. Participants least frequently searched for information about game on websites of the stores and from the restaurant service staff.

Only the frequency of using the three sources of information on game differed in the identified clusters, i.e., culinary programs, websites of the stores, and the restaurant service staff. The people in the “Indifferent” group searched for such information least often in culinary programs compared to others. The “Fearful” group, on the other hand, searched for information on game most often on websites of the stores and from the restaurant service staff (Table 5).

## 4. Discussion

Our study showed that the group of game consumers is not homogeneous, considering the perception of health risk factors associated with the consumption of game. Three participant groups were distinguished, namely people perceiving the highest health risk related to game consumption (“Fearful”), those who reported the risk associated with infection with parasites and zoonoses (“Selective”), and then people perceiving a low risk related to all factors (“Indifferent”). In turn, different perceptions of health risks showed a relationship for both attitudes towards game meat, and buying/eating behavior that ensure safety and mitigate health risks. The smallest differences between the selected clusters were noticed when opinions on the sources of information used to extend knowledge about the safety of game meat and its impact on health were included.

Similar to many studies showing that demographic variables, such as gender, age, and education, influence individuals’ food choice motives and behaviors [46,47], sociodemographic features were associated with the identified clusters. More men than women were represented in the “Indifferent” group, while more women than men were in the “Fearful” cluster. In the Italian population, men expressed higher confidence in the safety of game consumption [6], which could explain the greater proportion of men among the “Indifferent”. Other studies confirmed that women displayed health concerns more than men [48,49]; they are also more conscious of body image and appearance compared to men [50]. Such characteristics of women may explain the reporting of fears not only related to infection with parasites and zoonoses, but also related to gaining weight or increase in cholesterol level as a result of eating game.

Participants’ age also varied the perceived risk of game consumption. The youngest people were among the “Fearful” group, while the most people aged 55 and older were among the “Selective” group. Among the “Indifferent” group, there were the largest number of people aged 45–54.

More educated people reported greater concerns about health risks. The largest number of people with higher education was among the “Fearful” group, while the “Selective” cluster includes the respondents with secondary education mainly. A study conducted among Italian consumers [6] found that the cluster of “wild hunt game meat eaters”, which comprises people showing a high level of confidence in the safety of game meat, was characterized by a higher level of education.

Research shows that the knowledge based on the consumers’ own experiences significantly influences behavior towards food. Learning about food safety incidents leads to a relatively sudden and significant decrease in demand for potentially unsafe products [51]. The increase in anxiety often causes changes in the purchasing behavior, leading consumers to the resigning from products that cause concern and choosing food perceived as safe instead [52,53]. Trust in media and independent sources of information on food safety increases the perception of risk and thus reduces the likelihood of purchasing a product, while trust in public authorities has the opposite effect [54]. Direct and indirect experiences significantly influence the perception of risk. People who experience the food safety crises indirectly (e.g., because of a family member with a foodborne illness) have more concerns because the safety incidents have side effects, affecting not only the person experiencing the incident but also those in their social network [55].

In the opinion of all the respondents, regardless of the cluster classification, the most important threat related to the consumption of game is the possibility of contracting parasites and contracting a zoonotic disease. The greatest importance of these risks has been claimed by the 57.8% of the study sample (mainly the “Selective” and the “Fearful” cluster). Concerns are justified: many foodborne pathogens come from wildlife, which is evidenced by recent global disease outbreaks [1]. About 43% of human infectious diseases emerge from wildlife [56,57]. However, in the European countries, the microbiological quality of game meat has improved significantly as a result of good hunting practices and good hygiene practices [58].

Among the zoonoses, the particular importance of trichinosis should be exposed. The disease is most renowned and the most commonly diagnosed after the consumption of game meat in the area where the questionnaires were administered. It is caused by the contamination of meat with parasites of the family Trichinellidae in the larval form [59,60,61]. As the consumption of wild boar (*Sus scrofa*) meat is commonly associated by the consumers with trichinosis, the other risk factors, as bacteria contamination, may go unnoticed. However, the research shows that the bacteria contamination of wild boar meat is a greater threat to public health than pork [62].

The problem of trichinosis was common in Poland two decades ago, but as a result of coordinated actions, it was effectively resolved in 2006. The level of consumer protection against contracting trichinosis in the European Union is noted to be high [63]. Legal regulations systematically introduced in Europe resulted in a major reduction in the significance of risk. Regulation (EC) No. 853/2004 states: In order to ensure proper inspection of hunted wild game placed on the Community market, bodies of hunted animals and their viscera should be presented for official post-mortem inspection at a game-handling establishment [64].

In Poland, the mandatory veterinary testing for the presence of larvae in tissues [65] is an effective method to prevent trichinosis infection. However, in some regions, the safety standards and law regulations of the introduction of the game in the food market are sparse and do not guarantee proper consumer protection [1].

In Europe, it is also required for the hunters to have the proper knowledge and to be able to identify the main symptoms of infectious diseases during the primary inspection of the carcass, which is conducted right after the shooting [55]. Paradoxically, the trichinosis had been diagnosed mostly among the hunters, their relatives, and people related to them through professional and social relationships. The main cause of the cases was the consumption of meat that have not undergone veterinary control or proper culinary procedures [66,67,68]. Despite cases of disease among the hunter community, game meat derived directly from hunters is perceived as a high-quality product [69]. On the other hand, as the Italian study shows, consumers still have some concern towards hunters, believing that they ignore safety rules and do not follow hunting regulations [6].

The consumer’s belief in the safety of the game meat consumption can also be influence by information on African Swine fever (ASF) and Chronic Wasting Disease (CWD)—the diseases of wild and domestic animals that could have a high economic impact on farming [70]. Despite no evidence of the disease’s impact on humans, the reports of the outbreaks could lead consumers to associate them with zoonoses and therefore affects their behavior. However, risk cannot be ignored since it possible that the consumption of infected deer meat may be a source of infection of CWD [71]. Therefore, the number of cases of ASF and CWD should be subject to constant epidemiological supervision as part of the control and prevention programs of the foodborne risks [72,73].

One of the risk factors specific to game meat is the content of heavy metals in wild game tissues. The presence of heavy metals in game meat may be caused by the state of the environment [74], but also by the acquisition process, i.e., shooting with lead-containing ammunition [37,38,75]. However, research has proven that the level of particular elements usually did not exceed the content allowed by the standards [76,77]. Despite this, many countries recommend limiting the consumption of game by pregnant women and children because of the potential health risks [37,75,78]. It appeared, however, that the content of heavy metals in game meat was rated as least risky in the study sample. Only the “Fearful” were more afraid of consuming heavy metals, and at the same time, they were more likely not to accept hunting as a method of obtaining game.

The key to maintain high confidence in game and, at the same time, a low health risk associated with its consumption is therefore taking the right actions by game handlers (including hunters) and other participants in the sales chain, in line with accepted standards [5]. To ensure consumer safety, it is mainly recommended to use the proper heat-processing and suitable storage conditions [79,80]. However, the consumers’ safety would be endangered if the other stakeholders in the game market would not follow the regulations.

The study participants used various methods to lower the risk associated with game consumption. In addition to not consuming raw meat, they only eat game at home, eat only game tested by a vet, but also seek information about game. Nevertheless, the identified clusters showed their specificity in this field.

Most of the people in the “Selective” group ate game only at home, while most people in the “Indifferent” group bought/ate game tested by a veterinarian. The “Fearful” group, in turn, did not eat raw meat and sought information about game. For about 86% of the study sample, it was important to buy/eat game that has been tested by a veterinarian. At the same time, almost 95% of the “Indifferent” reported such behaviors, which may also explain their perception of the smallest health risk resulting from game consumption. Consumers also show readiness to pay more for meat of better quality, e.g., free from hormones, pesticides, and antibiotics [18,81]. That behavior has been noted mostly among the “Fearful” cluster.

Our research has shown that participants most often looked for information about the safety of game and its impact on health on product packaging (label) or at the seller. The “Selective” and “Fearful” groups were more likely to search for information about game on TV or radio culinary programs. The “Indifferent” group, on the other hand, were the ones using websites and restaurant service staff as sources of information. A previous study showed that the sources of information on game directly related to the industry and the product were trusted. In contrast, in the case of halal meat, for example, independent sources were mainly respected [82]. It should be emphasized that acquiring new information constantly evolves, along with the changing conditions of human life, as evidenced by the high assessment of the importance of the Internet. In the study sample, however, the Internet and family and friends were equally used as sources of information about the game meat.

It is noted that consumer reactions to media reports on food safety risks are different for various communities and products [83]. The food scandals may be an important factor leading to a reduction in the level of food safety perceived by consumers [84]. Our study proved that the need for new information about game is great, and especially for the “Fearful”; however, we did not measure reactions to such information. The example of the USA proves that information about the infection of meat products with pathogens (*Salmonella* and *Escherichia coli*) did not cause any significant changes in the structure of food consumption, while in case of a stronger consumer reaction, the changes in purchasing behavior were of short duration [85].

The publication of reports on the Creutzfeldt–Jakob disease risk from the consumption of beef containing BSE prions in cattle resulted in a significant drop in the consumption of this meat in Japan, while American consumers showed even increased interest in this product [86,87]. Canadian consumers, on the other hand, despite the declared high level of satisfaction with the functioning of food safety units, are concerned about the lack of transparency of the system and how food-related risks are communicated to the public [88].

The strength of our results is a relatively large sample of game consumers. However, our findings are specific to the Polish cultural background and should be used with caution in relation to other countries. Moreover, some other limitations should be considered when using the results. A cross-sectional study was used, which did not allow to assess the causality of relationships between the variables. Although findings should not be generalized to a Polish population and to populations with a different cultural background, our study provides an interesting insight into determinants of game consumption.

## 5. Conclusions

Despite the growing popularity of game, many aspects related to its consumption remain unexplored. In the context of this study, however, it should be pointed out that the perception of game consumers as a homogeneous group is a false assumption. The obtained results prove that people who consume game present different attitudes towards its safety, perception of threats and also declare different information needs. Differences between the groups of game consumers may be due to their own experiences, habits taken from the family home, and current conditions, such as the influence of the environment.

The implementation of educational and information activities may positively affect the perception of game by consumers, which may ultimately contribute to a change in the cluster structure of a given consumer group. It can also encourage meat producers to prepare a product that meets the expectations of potential buyers by highlighting not only its special features but also emphasizing safety and quality aspects.

## Figures and Tables

**Table 1:** Sociodemographic characteristics of the study sample and the identified clusters (%; *N* = 450, Poland).

Variables	Total Sample (*N* = 450)	Cluster 1 “Selective” (*N* = 125)	Cluster 2 “Indifferent” (*N* = 190)	Cluster 3 “Fearful” (*N* = 135)
Gender (*p* = 0.034 *)				
Women	41.3	42.4	38.9	56.3
Men	58.7	57.6	61.1	43.7
Age (years) (*p* = 0.033 *)				
25–34	37.1	32.0	34.7	45.2
35–44	27.1	26.4	28.9	25.1
45–54	24.7	23.2	27.9	21.5
over 55	11.1	18.4	8.5	8.2
Education (*p* = 0.048 *)				
Vocational	2.4	4.0	2.1	1.5
Secondary	77.3	83.2	77.4	71.8
Higher	20.3	12.8	20.5	26.7
Place of residence (*p* = 0.004 *)				
Rural area	45.3	49.6	51.1	33.3
Urban area	54.7	50.4	48.9	66.7
Professional situation (*p* = 0.014 *)				
I work full-time	75.6	68.8	74.2	83.7
I work part-time	6.4	11.2	6.3	2.2
I am a stay-at-home spouse/partner	10.7	9.6	11.1	11.1
I am at school/university	3.3	6.4	2.6	1.5
I am a pensioner	4.0	4.0	5.8	1.5
Household size (*p* = 0.046 *)				
1	4.2	4.8	3.2	5.2
2	15.6	16.0	16.3	14.1
3	34.4	32.8	31.6	40.0
4	30.2	34.4	32.1	23.7
5	12.0	8.0	13.1	14.1
>6	3.6	4.0	3.7	2.9
Self-reported financial situation (*p* < 0.001 *)				
There is not enough money even for basic needs	0.7	2.4	0.0	0.0
The money is enough for basic needs, but we cannot afford major expenses	4.2	7.2	4.2	1.5
We can afford everything, but we have to plan bigger purchases	38.9	44.8	38.4	34.1
We can afford everything and save some money	56.2	45.6	57.4	64.4

N—numbers of respondents; * *p*-value—Chi-square test.

**Table 2:** Clusters identified according to the participants’ opinions on health risk factors associated with the consumption of game (mean value ± SD *; *N* = 450, Poland).

Health Risks	Total Sample (SD)	SD	Cluster 1 “Selective” *N* = 125	Cluster 2 “Indifferent” *N* = 190	Cluster 3 “Fearful” *N* = 135
Infection with parasites	2.9	1.5	3.6 ^b^	1.4 ^c^	4.3 ^a^
Infection with zoonoses	2.6	1.4	3.2 ^b^	1.4 ^c^	3.9 ^a^
Increase in cholesterol level	2.2	1.2	1.9 ^b^	1.4 ^c^	3.5 ^a^
Gaining weight	2.1	1.1	1.7 ^b^	1.5 ^c^	3.3 ^a^
Intake of heavy metals remaining in meat after a shot	1.8	1.1	1.4 ^b^	1.3 ^b^	2.9 ^a^

* A five-point scale ranging from “there is no such risk” (1) to “the risk is serious” (5); SD—standard deviation; N—numbers of respondents, ^a,b,c^ different superscripts in each line indicate significant differences at *p* < 0.05 between identified clusters (ANOVA with post-hoc Waller–Duncan K-ratio, *t*-test, *p* < 0.05).

**Table 3:** Clusters’ profile according to the participants’ attitudes towards game meat (%; *N* = 450, Poland).

Statements	Total Sample (*N* = 450)	Cluster 1 “Selective” (*N* = 125)	Cluster 2 “Indifferent” (*N* = 190)	Cluster 3 “Fearful” (*N* = 135)
In my home, game dishes are prepared only for special occasions and festivities (*p* = 0.003) *	81.8	72.0	84.2	87.4
Game is a readily available product that can be found in many stores (*p* = 0.308)	23.8	20.8	27.4	21.5
I do not accept hunting as a method of obtaining game (0.035)	82.7	82.2	78.4	92.8
I took the tradition of eating game meat from my family home (*p* < 0.001)	11.3	6.4	20.0	3.7
Game meat and products are value for money (*p* = 0.014)	76.0	83.2	69.5	78.5
I value game meat for its natural origin (*p* = 0.045)	88.9	88.8	86.3	92.6
Game meat has a positive effect on my health (*p* = 0.042)	77.6	75.2	83.1	71.9

N—numbers of respondents; * *p*-value—Chi-square test.

**Table 4:** Cluster profiles according to the participants’ buying and eating behavior that ensure safety and mitigate health risks (*N* = 450, Poland).

Statements	Total Sample (*N* = 450)	Cluster 1 “Selective” (*N* = 125)	Cluster 2 “Indifferent” (*N* = 190)	Cluster 3 “Fearful” (*N* = 135)
I eat game only in a trusted eating place (*p* = 0.016) *	52.0	62.4	50.0	45.1
I only buy/eat game that has been tested by a veterinarian (*p* < 0.001)	86.2	82.4	94.7	77.8
I only buy game from a reliable source (*p* < 0.001)	62.9	68.0	49.5	77.1
I am willing to pay more for better quality meat (*p* < 0.001)	79.3	72.8	74.7	91.9
I only eat game at home (*p* = 0.005)	86.4	92.8	80.5	88.9
I do not eat raw meat (*p* = 0.046)	95.1	93.6	92.6	97.9
I seek information on game (*p* = 0.024)	84.2	83.2	80.0	91.1

N—numbers of respondents; * *p*-value—Chi-square test.

**Table 5:** Cluster profiles according to the participants’ opinions on using the sources of information about the game meat (mean value ± SD *; *N* = 450, Poland).

Sources of Information (from…)	Mean	SD	Cluster 1 Selective *N* = 125	Cluster 2 Indifferent *N* = 190	Cluster 3 Fearful *N* = 135	*p*-Value
Culinary programs (e.g., on TV, in radio)	3.0	0.8	3.2 ^a^	2.8 ^b^	3.1 ^a^	0.0015 *
Internet (e.g., food portals)	3.3	1.1	3.2 ^a^	3.3 ^a^	3.5 ^a^	0.1275
Producers’ websites	2.9	1.0	2.9	2.8	2.8	0.2047
Websites of the stores	2.7	0.9	2.6 ^b^	2.6 ^b^	2.9 ^a^	0.0273 *
Family and friends	3.3	0,9	3.4	3.3	3.4	0.0966
Doctors/nutrition specialists	3,2	0.8	3.2	3.2	3.3	0.4188
Sellers	3.5	0.8	3.5	3.5	3.6	0.2060
Labels on the packaging	3.5	0.9	3.5	3.5	3.6	0.3073
Advertising (e.g., TV, press, billboards)	3.2	0.7	3.2	3.1	3.2	0.2090
Store leaflets	3.3	1.3	3.1	3.4	3.3	0.0937
Press articles	3.2	0.9	3.0	3.2	3.2	0.2429
Culinary books	2.9	1.1	2.9	2.9	3.1	0.3094
Restaurant service staff	2.7	1.0	2.6 ^b^	2.6 ^ab^	2.8 ^a^	0.0474 *

* A five-point scale ranging from “I do not use this source at all” (1) to “I use this source most frequently” (5); SD—standard deviation; N—numbers of respondents, ^a,b^ different superscripts in each line indicate significant differences at *p* < 0.05 between identified clusters (ANOVA with post-hoc Waller–Duncan K-ratio *t*-test, *p* < 0.05).
